# The comparison of alternative splicing among the multiple tissues in cucumber

**DOI:** 10.1186/s12870-017-1217-x

**Published:** 2018-01-05

**Authors:** Ying Sun, Han Hou, Hongtao Song, Kui Lin, Zhonghua Zhang, Jinglu Hu, Erli Pang

**Affiliations:** 1 MOE Key Laboratory for Biodiversity Science and Ecological Engineering, College of Life Sciences, Beijing Normal University, No 19 Xinjiekouwai Street, Beijing, 100875 China; 2 Beijing Key Laboratory of Gene Resource and Molecular Development, College of Life Sciences, Beijing Normal University, No 19 Xinjiekouwai Street, Beijing, 100875 China; 3 Key Laboratory of Biology and Genetic Improvement of Horticultural Crops, Ministry of Agriculture, Institute of Vegetables and Flowers, Chinese Academy of Agricultural Sciences, Beijing, 100081 China; 4 Tobacco Research Institute of Chinese Academy of Agricultural Sciences (CAAS), Qingdao, 266101 China; 5 Graduate School of Information, Production and Systems, Waseda University, Kitakyushu-shi, 808-0135 Japan

**Keywords:** Alternative splicing, Tissues, Tissue-specific, Cucumber

## Abstract

**Background:**

Alternative splicing (AS) is an important post-transcriptional process. It has been suggested that most AS events are subject to tissue-specific regulation. However, the global dynamics of AS in different tissues are poorly explored.

**Results:**

To analyse global changes in AS in multiple tissues, we identified the AS events and constructed a comprehensive catalogue of AS events within each tissue based on the genome-wide RNA-seq reads from ten tissues in cucumber. First, we found that 58% of the multi-exon genes underwent AS. We further obtained 565 genes with significantly more AS events compared with random genes. These genes were found significant enrichment in biological processes related to the regulation of actin filament length. Second, significantly different AS event profiles among ten tissues were found. The tissues with the same origin of development are more likely to have a relatively similar AS profile. Moreover, 7370 genes showed tissue-specific AS events and were highly enriched in biological processes related to the positive regulation of cellular component organization. Root-specificity AS genes were related to the cellular response to DNA damage stimulus. Third, the genes with different intron retention (IR) patterns among the ten tissues showed significant difference in GC percentages of the retained intron, and the number of exons and FPKM of the major transcripts.

**Conclusions:**

Our study provided a comprehensive view of AS in multiple tissues. We revealed novel insights into the patterns of AS in multiple tissues and the tissue-specific AS in cucumber.

**Electronic supplementary material:**

The online version of this article (10.1186/s12870-017-1217-x) contains supplementary material, which is available to authorized users.

## Background

Alternative splicing (AS) is an important post-transcriptional process by which multiple transcripts are generated from a single gene. It plays a key regulatory role in modulating the transcriptome and proteome diversity [1–4]. Moreover, AS can serve other regulatory functions in development, tissue, and species specificity [5–7]. Specifically, plants are always exposed to environmental stress and are regulated by many plant processes through differential splicing [8]. With the development of sequencing technologies, AS in plants is coming of age [9].

AS was first proposed by Walter Gilbert in 1978 [10] and has been reported in several genes [11, 12]. AS is now highly pervasive in eukaryotes. AS events are classified into four basic types depending on the regions affected: intron retention (IR) events, exon skipping (ES) events, alternative donor site (AD) events, and alternative acceptor site (AA) events [13]. IR events are those where an intron is not spliced out but instead combines with the flanking exons to form a longer exon instead. The ES events refer to a whole exon spliced out along with its flanking introns. AD and AA events are those where the alternative 3′ or 5′ boundary is used. Among the AS events, ES is the most common type in metazoans [14], whereas IR is the most common type of AS in plants [15].

AS was generally estimated from the transcriptome data at the genome-wide level; therefore, transcriptome data must be obtained to assess AS. Over the past decades, three main sequence-based technologies were used to obtain these data: expressed tags (ESTs), microarrays and RNA sequencing (RNA-seq). ESTs are 200–800 nucleotide bases in length. They are randomly selected single-pass sequence reads, so they are subject to sampling bias [16]. AS events were identified from 40 to 60% [17, 18] human genes and 22% Arabidopsis genes [15] deduced from ESTs. Microarrays are highly throughput but require prior knowledge of the genome sequence. Microarray-based AS databases indicated that at least 74% of human multi-exon genes underwent AS [19]. However, RNA-seq has revolutionized the manner in which eukaryotic transcriptomes [20] were obtained, which allowed for a systematic, unbiased inquiry of the transcriptome. Recently, based on RNA-seq data, 95% human genes [21] and 61% Arabidopsis genes [4] were reported to produce more than one transcript through AS.

Since on the advent of RNA-seq technology, the estimates of AS have increased considerably, indicating their primary dependence on the amount and coverage of the transcripts from an organism [22], researchers tried to increase the amount and coverage of transcripts to better understand AS. In human, 15 tissues and cell line transcriptomes were reported, and over 50% AS isoforms were found to be differentially expressed among tissues [23]. This indicated that most AS is subject to tissue-specific regulation [24]. In plants, recent transcriptome-wide analysis of AS using RNA-seq reads from different tissues, and developmental stages were also reported. In soybean, RNA-seq reads from 28 developing tissues revealed that gene structure and genomic features were the main factors affecting AS frequency [25]. In maize, two studies explored AS in different tissues and stress conditions. RNA-seq reads from a variety of tissues in two different genotypes of maize were obtained, which demonstrated that many genes encoding novel transcripts were often expressed in a tissue-specific manner [26]. RNA-seq reads from ear, tassel and leaves of maize under both well-watered and drought conditions demonstrated that AS is strongly associated with the tissue type, development stage and stress condition [27]. A deep survey of AS in grape from different genotypes under different tissues and stress conditions showed that AS was correlated to tissue types and genotypes [28]. RNA-seq reads from seedlings, flowers and early growth fruits in tomato revealed that more splice variants per gene were generated in early growth fruits [29]. These studies further revealed that AS in plants is far more complex than previously observed [22].

Cucumber (*Cucumis sativus* var. *sativus*) is one of the most economically important cultivated plants. The genome sequence of cucumber was reported in 2009 [30]. The RNA-seq reads from 10 tissues were sequenced to improve genome annotation [31]. Nevertheless, the landscape of AS and difference of AS among multiple tissues have not been explored in cucumber. Thus, using the genome-wide RNA-seq reads from 10 tissues in cucumber, we provided an overview of AS and then analysed AS at the tissue level. We found that 58.17% of the multi-exon genes underwent AS. The AS event profiles were found to be significantly different among the ten tissues in cucumber. Moreover, stem and leaf tissues showed relative similar AS profile. We also found 7370 genes with tissue-specific AS among the ten tissues. This collection will be a valuable resource for further research of the AS function in plants.

## Methods

### Data sources

In this study, we mainly used three types of data: genome sequence, genome annotation and RNA-seq reads. The genome sequence and genome annotation (version 2) of cucumber were downloaded from the following website: http://cmb.bnu.edu.cn/Cucumis_sativus_v20/ [30]. All these data were from *Cucumis sativus* var. *sativus* line 9930. Moreover, the RNA-seq reads of ten tissues were collected from the SRA database (https://www.ncbi.nlm.nih.gov/sra/) (SRA: SRA046916) [31]. These tissues included ovary, expanded ovary under fertilization (7 days after flowering), expanded ovary not fertilized (7 days after flowering), root, stem, leaf, male flower, female flower, tendril, and base part of tendril. These sequences are 75-nt paired-end sequencing reads.

### Reads pre-processing

The quality of raw reads was assessed using FastQC (version 0.11.2) [32]. Then, the low-quality bases were removed using Trimmomatic (version 0.35) [33] with default parameters. Reads with length equal or more than 25 bp on both sides of paired-end format were kept for further analysis.

### Transcriptome assembly using reference genome-based reads mapping

To obtain the cucumber transcripts, we aligned all high-quality reads to the reference genome using TopHat (version 2.1.0) (parameters ‘-N 5 –read-gap-length 3 –read-edit-dist 5’) [29, 34, 35] and Cufflinks (version 2.2.1) (parameters -b –u -g) [36, 37]. The abundance of the transcripts was measured by FPKM (expected number of fragments per kilobase of transcript sequence per millions base pairs sequenced). Transcripts with FPKM lower than 0.1 were filtered out [29], and junction-sites were extracted by RSeQC (version 2.6.4) [38].

### Identification of alternative splicing events

To identify AS events, the assembled transcript isoforms were mapped to the reference genome structure annotation using Cuffcompare. Then, we acquired a non-redundant transcript pool [36]. The isoforms encoded by annotated genes were extracted and used for the next analysis. ASTALAVISTA, a web server that can extract AS events as much as possible, was used to detect AS events [39]. Four basic types of AS events, including IR, ES, AD and AA, were further analysed.

### Construction of the AS event profiles of tissues

To compare the AS events among the ten tissues, we first constructed a vector P = (N_1_,N_2_,N_3_,N_4_) representing the AS event profile for each tissue, where N_i_ (i∈1,..,4) was the number of the i-th class AS events in one tissue. The four classes of AS events were IR, AA, AD and ES.

### Construction of the IR patterns of genes

First, all genes underwent IR events in the ten tissues were extracted. Second, we constructed a vector P_IR_ = ({N_i_}), where N_i_(i∈{1,..,10}) was 1 if occurring IR in the i-th tissue or was 0 if not occurring IR, which represented the IR pattern of a gene. The ten tissues are ovary, expanded ovary under fertilization, expanded ovary not fertilized, root, stem, leaf, male flower, female flower, tendril, and base part of tendril.

### Hierarchical clustering analysis

To compare the similarity of AS event profiles among the tissues, the profiles of ten tissues were clustered based on the distances using hierarchical clustering [40]. According to the number of these four basic types of AS events among the ten tissues, a plot was drawn with the complete linkage. To detect the similarity among tissues, a relative distance was used.

Since IR was the common AS type in plants, what features may the genes with the similarity of IR patterns have? To answer the question, the IR patterns of genes were clustered using hierarchical clustering. The index to measure the distance was a binary metric.

### Functional annotation of genes and gene ontology enrichment analysis

To annotate the reference genes, the sequences of annotated genes were used as queries in the BLASTX searches against the Swiss-Prot database, using the Blast2GO with default parameters [41]. Then, Gene Ontology (GO) terms were assigned for annotated genes. Enrichment analysis of GO was conducted using the tool Ontologizer with a background of expressed genes [42]. The Benjamini-Hochberg method was performed for multi-test correction. Over-represented functional GO terms were selected for those with a false discovery rate (FDR) smaller than 0.05.

### Randomization process

To detect the genes with significantly more AS events than expected with random genes, we followed a randomization method. For each AS gene, we got the number of AS events as the observed value. Then, we randomly extracted one gene from the other AS genes. This randomization process was repeated 1000 times. We can obtain an empirical *p-value* for each AS gene. The *p-value* was defined as how often these numbers of AS events are greater than the observed value. If the *p-value* of a gene was less than 0.05, the occurrence of AS events was more significant than those expected at random.

### Identification of genes showing tissue-specific AS among the ten tissues

AS genes may contain multiple AS events, which may not occur simultaneously in the ten tissues. Moreover, some of AS events were only found in individual tissue. If a gene contains an AS event in only one tissue among the ten tissues, it was defined as a gene with a tissue-specific AS event.

### RT-PCR validation of AS events

*Cucumis sativus var. sativus* line 9930, which is from Institute of Vegetables and Flowers, Chinese Academy of Agricultural Sciences (IVF-CAAS), was used in this experiment. The plants were grown under long-day conditions (16/8-h day/night cycle at 25 °C/15 °C) with permissions from the local government. Top leaves and stems were harvested from the 30-Day plants under the institutional guildline of IVF-CAAS, and then flash-frozen in liquid nitrogen. Total RNA was extracted by Trizol reagent (Tiangen, China). RT-PCR was used to produce the first cDNA strand by Fast Quant RT Kit (Tiangen, China). DNA were reproduced by 30 cycles of PCR, and analyzed by PAGE gel. 

### Statistical tests

Fisher’s one-tailed test was used for the analysis of enrichment. Chi-square test was used to analyse the differences in AS event profiles among the ten tissues. Mann-Whitney U-test was used to compare the difference in lengths between the retained and non-retained introns. Kruskal–Wallis rank sum test was used to analyse the differences among multiple groups. All statistical tests were performed using the R statistical package.

## Results

### Overview of the transcriptome

To explore the AS events, we downloaded 75-nt paired-end RNA-seq reads from the ten tissues, including ovary, expanded ovary under fertilization, expanded ovary not fertilized, root, stem, leaf, male flower, female flower, tendril, and the base part of the tendril. We applied Trimmomatic to filter out the low-quality reads [33]. After quality control of the reads, 199,307,425 high-quality reads, occupying 90.39% of the total reads, were remained (Table 1).

**Table 1:** Summary of RNA-seq read counts and mapping statistics

Tissue	Raw reads	High-quality reads	Mapped reads	Unique mapped reads
Ovary	19,247,768	16,705,294	15,639,192	14,660,009
Expanded ovary under fertilization	18,466,067	16,438,206	15,290,470	14,273,961
Expanded ovary not fertilized	19,111,746	16,401,257	15,198,753	13,925,987
Root	18,732,466	16,378,025	15,275,644	14,117,427
Stem	24,535,215	22,825,990	21,248,383	19,439,971
Leaf	26,400,675	24,731,604	22,568,880	20,738,393
Male flower	26,050,858	24,438,476	22,788,799	21,120,178
Female flower	23,818,868	21,725,618	19,906,003	18,627,983
Tendril	22,472,146	20,302,220	18,789,694	17,743,431
Base part of tendril	21,653,855	19,360,735	17,902,815	16,919,102

We used the program TopHat2 [34] to map the high-quality reads to the cucumber genome, in which 92.94% reads were uniquely aligned (Table 1). Moreover, we obtained 36,910 complete novel junctions and 40,884 partial novel junctions (Additional file 1: Figure S1a), which can help in finding new splicing events.

The RNA-seq reads mapped to the genome were assembled into transcripts using Cufflinks [36]. Altogether, 21,387 genes, corresponding to 88.11% (21387/24274) of the annotated genes, produced 74,543 transcripts, and were expressed in at least one of the ten tissues. These transcripts were distributed in ten tissues, ranging from 24,406 (the base part of tendril) to 27,175 (expanded ovary not fertilized) (Table 2). The number of expressed genes in the ten tissues ranged from 17,859 (tendril) to 18,572 (root) (Table 2). Moreover, 3344 new genes were detected, which produced 5173 transcripts.

**Table 2:** Overview of transcripts from the Cufflinks assembly

Tissue	Transcripts	Transcripts encoded by annotated genes	Genes annotated
Ovary	39,133	26,515	18,373
Expanded ovary under fertilization	38,777	26,272	18,308
Expanded ovary not fertilized	39,255	27,175	18,485
Root	38,442	26,421	18,572
Stem	36,900	24,565	17,895
Leaf	38,176	25,666	18,243
Male flower	38,336	25,970	18,332
Female flower	38,879	26,491	18,478
Tendril	36,144	24,473	17,859
Base part of tendril	35,973	24,406	17,877

### A global view of the AS events

**Fig. 1:**
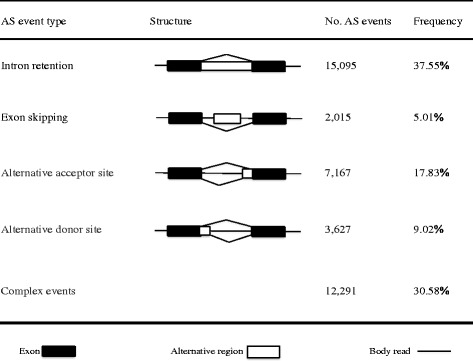
Statistics of different AS events

We applied the program ASTALAVISTA [39] to identify the AS events in cucumber. According to the current annotation, we analysed only the AS events occurring on the transcripts encoded by the annotated genes. A total of 40,195 AS events, distributed in 10,015 intron-containing genes, were identified, which accounted for 58.17% (10,015/17,216) of the expressed multi-exon genes in the ten tissues. Among the AS events identified, IR represented 37.55% of the total AS events and was the most abundant type, followed by AA (17.83%), AD (9.02%) and ES (5.01%) (Fig. 1). The results were consistent with the observations in soybean [25] and tomato [29]. In addition to the four basic types of AS events, there were also 12,291 complex AS events (30.58%), containing more than one of the four types of AS events, which further suggested the complexity of AS in cucumber (Fig. 1). Of all the AS events, 13,154 were identified by comparing the transcripts among different tissues (among-tissue events), and the other 27,041 were identified by comparing within individual tissues (within-tissue events). For the basic AS types, the ratios between within-tissue events and among-tissue events were similar: IR was the most common, whereas ES was the lowest (Additional file 2: Figure S2).

### Functional annotation of genes

To determine the functions of genes in cucumber, we conducted a functional annotation of all genes by performing Blast2GO [41]. Altogether, 59% (14,390/24,274) genes have potential functions, involving 8660 GO terms, which are partitioned into 5352 biological process, 2406 molecular functions and 902 cellular components.

### Genes subjected to AS

Of the annotated genes, 10,015 genes contained one or more AS events. To assess the potential functional relevance of the AS genes, we examined the functional associations of these genes. We tested for enrichment among the genes. Biological processes of AS genes were mainly enriched in the regulation of cellular component organization, protein modification process and cellular response to DNA damage stimulus. For molecular function, these genes were mainly enriched in N-acetyltransferase activity, endodeoxyribonuclease activity, transcription factor binding, and protein binding (Additional file 3: Figure S3). These results suggested the importance of AS genes in plants [43].

**Fig. 2:**
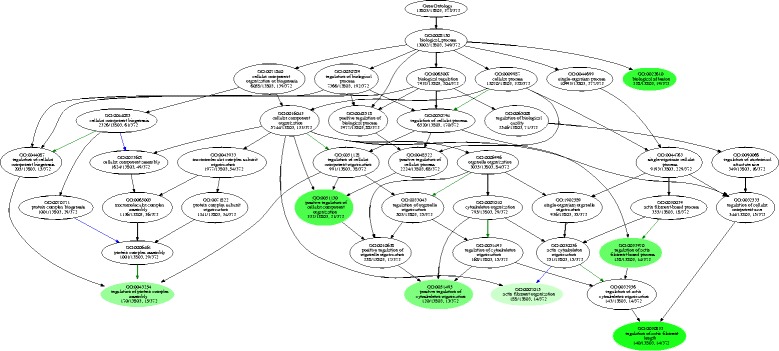
GO enrichment of genes with more AS events

For 10,015 AS genes, the number of AS events varied widely. Some genes were subjected to many AS events, whereas others were subjected to only a few. To gain further insight into the genes with significantly more AS events compared with random genes, a randomization process was used to extract these genes. We obtained 565 genes with significantly more AS events compared with random genes. We found that these genes were significantly enriched in biological processes related to regulation of actin filament length, such as positive regulation of cellular component organization, regulation of protein complex assembly, and biological adhesion (Fig. 2). A previous study reported that the rich AS of cell adhesion molecules increases the number of available cell–cell recognition molecules in the insect or vertebrate genome [44]. These results indicated that cell adhesion molecules might employ the similar mechanisms to increase the ability of cell–cell recognition molecules in cucumber.

### Four basic types of AS events

**Fig. 3:**
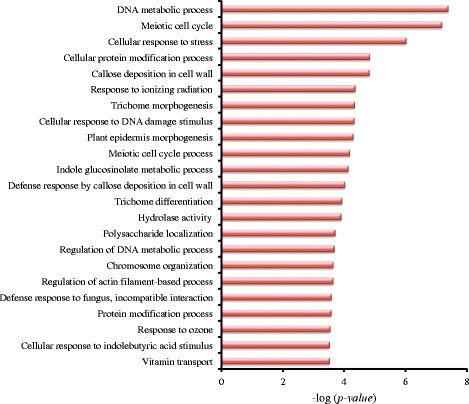
GO enrichment of genes with only IR events

For each type of AS events the mechanism of regulation is different. Therefore, we explored whether there is a functional significance for the different types of AS events occurring in different genes. As previously mentioned, there were 10,015 genes with AS, wherein the numbers of IR, AA, AD, and ES genes were 7241, 4558, 2648, and 1504, respectively. The GO enrichments showed that different AS gene types were involved in different functions, with a few overlapping GO terms (Additional file 4: Figure S4). The AS genes underwent different types of events simultaneously. To better understand the functions of the AS type, we isolated the genes with only one type of AS. We extracted 3262, 1153, 463 and 202 genes which only had IR, AA, AD and ES, types of events respectively. In genes with only the IR events, the significant biological processes were related to cell wall macromolecule metabolism (Fig. 3). In Arabidopsis, a gene regulated starch metabolism by IR type of AS [45]. This indicates that genes participating in regulating metabolism by IR might be widespread in plants.

### Different AS events profiles among the ten tissues

**Fig. 4:**
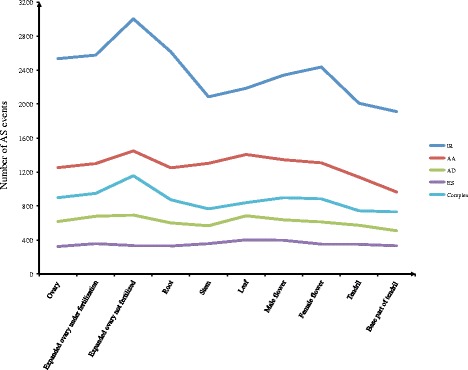
Numbers of different AS event types in the ten tissues

To further investigate the profiles of AS events in the ten tissues, we used the within-tissue events for further analysis. Among these AS events, IR was the most abundant type (1912–3004), followed by AA (966–1450), complex events (731–1157), AD (510–695) and ES (323–401) (Fig. 4). In the ten tissues studied, the highest AS events were found in the expanded not fertilized ovary, which were significantly higher than the expanded ovary under fertilized (Fisher’s one-tailed test, *p-value* = 1.53e-10). Conversely, the smallest number of AS events was detected in the base part of the tendril and was significantly lower than the tendril (Fisher’s one-tailed test, *p-value* = 2.48e-4) (Additional file 1: Figure S1b). This is in accordance with our hypothesis that the greater the number of transcripts, the higher the number of AS events; this result has been verified in human [46].

**Fig. 5:**
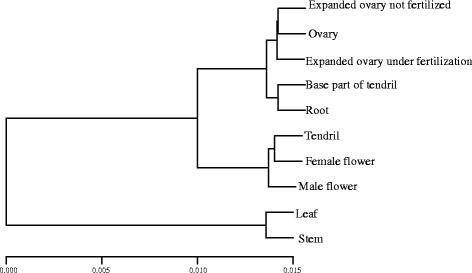
The cluster of AS event profiles of ten tissues

To compare the AS events among tissues, each tissue was represented by a vector denoted as an AS event profile. We found significantly different AS event profiles among the ten tissues (chi-square test, *p-value* ≤ 2.2e-16). To further detect the similarity in the AS event profiles among tissues, we used hierarchical clustering to analyse the relationship among the ten vectors. As Fig. 5 shows, the AS event profiles of stem and leaf are similar; the AS event profiles of the ovary, expanded ovary under fertilization, and the expanded ovary not fertilized were similar.

### Genes showing tissue-specific AS among the ten tissues

Many AS events are regulated in a tissue-specific manner; therefore, we wanted to determine the genes with tissue-specific AS among the ten tissues and their functions. We first isolated the genes showing tissue-specific AS event using our Perl scripts. Altogether, we observed 7,370 genes showing tissue-specific AS events, scattered in ten tissues. To understand the biological significance of these genes, the GO enrichment was applied with Ontologizer [42]. In the list of the genes with tissue-specific AS events among the ten tissues, we found that they were highly enriched in biological processes related to positive regulation of cellular component organization and molecular functions related to structure-specific DNA binding (Additional file 5: Figure S5). The results indicated that these genes might be involved in tissue-specific regulation.

We further investigated the genes with tissue-specific AS events. Among the ten tissues, the number of genes with tissue-specific AS events ranged from 1067 to 1763. The most genes with tissue-specific AS events were in the expanded ovary not fertilized, followed by root (1469) with significantly fewer genes with tissue-specific AS event than in the expanded ovary not fertilized (Fisher’s one-tailed test, *p-value* = 1.523e-06). The fewest genes with tissue-specific AS event were in the stem, followed by the base part of the tendril (1173) wherein there were significantly more genes with tissue-specific AS event than in stem (Fisher’s one-tailed test, *p*-value = 1.95e-02) (Additional file 1: Figure S1c).

**Fig. 6:**
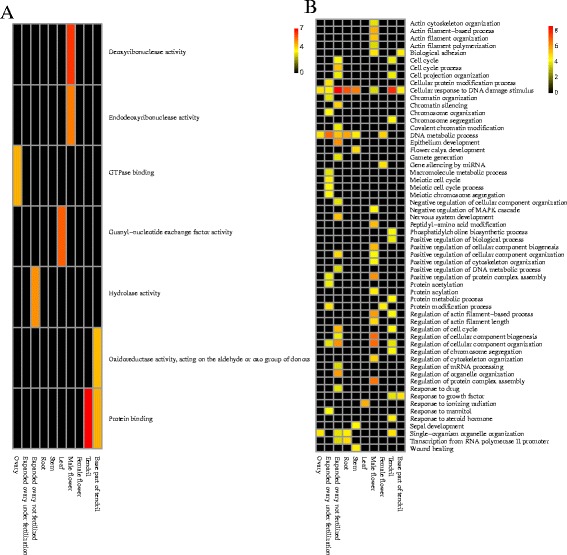
GO enrichment analysis of genes with tissue-specific AS events in ten tissues. The colour square represents $-{\log}_{10}^{p- value}$ for the enrichment of GO terms. Red indicates that the term is significantly enriched, and black represents no enrichment in the term. **a** Molecular function. **b** Biological process

The functions of the genes, with tissue-specificity for AS events in the ten tissues, were investigated using the GO enrichment results shown in Fig. 6. For example, in the root, the cellular response to DNA damage stimulus was significantly enriched. A previous study detected a stem cell niche in the Arabidopsis roots [47]. These stem cells must have effective DNA damage response to prevent mutations propagated to other parts of the plant [48]. Moreover, RNA splicing was a new player in the DNA damage response [49]. Based on these studies, we speculated that in cucumber roots there was a stem cell niche and root-specific AS events were observed in response to DNA damage.

### Genes with similar IR patterns among the ten tissues

IR is the dominant AS in plants, and short introns are more retained than long introns [25]. Therefore, we investigated the length of the retained introns compared with other introns and found that the lengths of the retained introns were significantly shorter than the other introns (Mann-Whitney U test, *p-value* ≤ 2.2e-16) (Additional file 6: Figure S6). This indicated a tendency for shorter retained introns in cucumber.

**Fig. 7:**
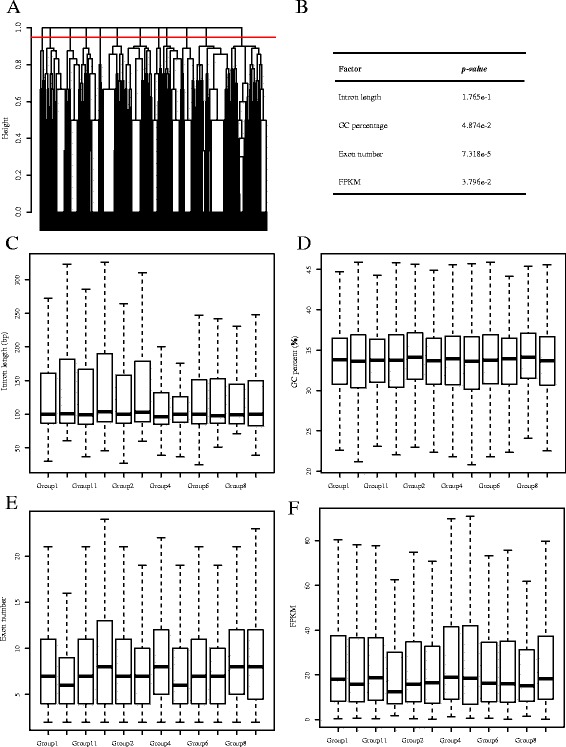
Clustering of genes by IR patterns. **a** The hierarchical clustering of IR genes. The red line was drawn at the height of 0.95, and these genes were classified into 12 parts. **b** Features of IR genes and *p-values* by Kruskal–Wallis rank sum test. **c** The length of the retained introns. **d** GC percentage of the retained introns. **e** Exon number of genes. **f** FPKM of major isoforms

For each gene undergoing IR, we obtained an IR pattern among the ten tissues (see Materials and Methods). According to their IR patterns, the genes were clustered into 12 classes (Fig. 7a) with genes in the same cluster having similar IR patterns. Based on this cluster, we explored 4 features of these 12 groups: retained-intron length, retained-intron GC percentage, the exon number and major transcripts (transcript with highest FPKM in all tissues for one gene) expression value. Interestingly, these groups had similar lengths for the retained introns (Kruskal–Wallis rank sum test, *p-value* = 0.1765); GC percentages of the retained introns, number of exons and FPKM of major transcripts are significantly different among the groups (Kruskal–Wallis rank sum test, *p-value* = 0.04874, *p-value* = 7.318e-05, *p-value* = 0.03796, respectively) (Fig. 7). These results indicated that the feature of genes might affect the IR patterns of genes in the ten tissues.

### Validation of AS events by RT-PCR

To validate the AS events predicted by RNA-seq, we performed reverse transcription-PCR (RT-PCR) experiments for 41 AS events in two tissues (leaf and stem) using newly extracted RNA. First, we verified the accuracy of these events in leaf, and 31 AS events (76%) were validated (Additional file 7: Figure S7). Then, we chose 18 out of 41 AS events, which showed different AS events between leaf and stem. Among these 18 events, 10 (56%) were validated (Additional file 8: Figure S8). Due to the sensitivity of RT-PCR, more events can be obtained. Given that the RNAs for the experiments are not the same for generating the analyzed RNA-seq, some AS events which were not validated may be due to the changes of spatial and temporal gene expression. Furthermore, according to the results detected by the gel, we can see the differences in expression levels between the primary and alternative transcript, such as Csa5G621964 and Csa6G01160 (Additional file 8: Figure S8).

## Discussion

In this study, we conducted a systematic analysis of the transcriptome of cucumber using RNA-seq data. Our results significantly increased the complexity of transcripts; specially, no isoforms were detected in cucumber V2.

AS plays a key role in plant development and stress adaptation [26]. Previous studies attempted to explore the relationship between AS and development and between AS and stress conditions. In soybean and tomato, AS events have different distributions among different developmental stages, and younger developmental stages have a higher frequency of AS genes [25, 29]. In *Zea mays*, it was verified that the different isoforms expressed in different tissues might gain or lose functional domains, and the different expressions of trans-acting factors are more likely to cause tissue-specific events [27]. In grape, the relationship of the tissue, stress and genotype with AS were studied, and the result showed that tissues are important factors affecting AS [28]. In our study, we focused on the profiles of AS events among the ten tissues and obtained the genes with more AS events compared to random genes. Moreover, we identified tissue-specific AS events based on the sites of AS, compared the IR patterns of genes and explored the relationship between IR patterns and features of genes.

Our analysis was based on the RNA-seq data; therefore, the amount and diversity of the transcriptome data would affect our identification of AS events. To ensure that the sequencing depth for each tissue was sufficient to perform alternative splicing analyses, we extracted a re-sampled subset of reads by RSeQC [38] and found the number of detected splicing junctions was close to the fixed value with an increase in the resample percentage increasing (Additional file 9: Figure S9). This indicated that the depth of reads for each tissue was sufficient for AS analysis.

Based on the RNA-seq data from the ten tissues, 40,195 AS events were extracted overall, involved nearly 60% multi-exon genes. By comparing the four basic types of AS events, we found that the predominant type of AS is IR, and ES only accounts for a small proportion. This result was similar to those with other plants [50]. Moreover, the genes showing more AS events appeared to be related to the actin filament. Previous studies showed that the actin filament is the main factor for polarized plant cell growth [51] suggesting that more splicing variations are required by the plant growth.

In our study, the AS event profiles were significantly different among the ten tissues. We further analysed two flowers, three ovaries, stem and leaf that showed relatively similar profiles for AS events. The origin of the stem and leaf from the same germinal cells might explain the similarity. The separate tendrils also imply the complexity of AS among tissues. Furthermore, as IR was the dominant AS type in plants, we suggested that the features of genes might affect their IR patterns among tissues.

The advances in RNA-seq technology provide a great opportunity for studying AS. For instance, the percentage of AS gene in Arabidopsis increases significantly from 1.2% to 61% [4, 52]. In cucumber, the AS events were studied by EST data at the genome-wide level, and 430 events were identified [53]. In this study, we observed 40,195 AS events nearly 100 times higher than before. Sequencing technologies provide more data and longer transcripts, which provide a better opportunity to study AS [29].

## Conclusions

In summary, using RNA-seq reads, we identified many AS events from the ten tissues in cucumber. This collection will be a valuable resource for further research of AS. Moreover, we found that the ten tissues showed significantly different AS event profiles and tissue-specific events. These results will promote our understanding of AS in different tissues and elucidate the patterns of AS events among different tissues. Moreover, our results pave the way for future functional studies on transcripts forms of cucumber.

## Additional file

Additional file 1: Figure S1. AS events of ten tissues. (a) Numbers of junctions. The splice sites were divided into the 5′splice site (5’SS) and 3′splice site (3’SS). The dark blue colour represents the number of junctions with two splice sites have been annotated in genome annotation. The green colour represents the number of junctions whose two splice sites are novel in genome annotation. The orange colour represents the number of junctions whose splice sites were found in genome annotation. (b) Number of AS events. (c) Number of genes with tissue-specific AS. (PDF 766 kb)
Additional file 2: Figure S2. AS events of within-tissue and among-tissue. (a) The total number of 4 basic types of AS events for among-tissue and within-tissue. (b) Basic types of AS events among tissues. (c) Basic types of AS events within tissues. (PDF 488 kb)
Additional file 3: Figure S3. GO enrichment of AS genes. Only the top 30 significantly enriched GO terms are listed. (a) Molecular function. (b) Biological process. (PDF 298 kb)
Additional file 4: Figure S4. GO enrichment analysis of the basic AS types. Colour squares represent $-{\log}_{10}^{p- value}$ for the enrichment of GO terms. Red means that the term is significantly enriched, and black represent no enrichment in this term. Only significant enrichment GO terms of each type were listed. (a) Molecular function. (b) Biological process. (PDF 969 kb)
Additional file 5: Figure S5. GO enrichment of genes with tissue-specific AS. Only the top 30 significant enriched GO term were listed. (a) Molecular function (b) Biological process. (PDF 305 kb)
Additional file 6: Figure S6. The lengths of introns between the retained introns and not retained introns. (PDF 102 kb)
Additional file 7: Figure S7. Verify of AS events by RT-PCR in leaf. (PDF 759 kb)
Additional file 8: Figure S8. Verify of AS events by RT-PCR in leaf and stem. (PDF 2393 kb)
Additional file 9: Figure S9. Saturation analysis of junction detection. (a) Ovary. (b) Expanded ovary under fertilization. (c) Expanded ovary not fertilized. (d) Root. (e) Stem. (f) Leaf. (g) Male flower. (h) Female flower. (i) Tendril. (j) Base part of tendril. (PDF 570 kb)

## Abbreviations

AA: Alternative acceptor site
AD: Alternative donor site
AS: Alternative splicing
ES: Exon skipping
ESTs: Expressed tags
FPKM: Expected number of fragments per kilobase of transcript sequence per millions base pairs sequenced
IR: Intron retention
RNA-seq: RNA sequencing
RT-PCR: Reverse transcription-PCR
