# Supplementary figures and images for: The comparison of alternative splicing among the multiple tissues in cucumber
Source: BMC Plant Biol. 2018 Jan 5;18:5. doi: 10.1186/s12870-017-1217-x (PMC5755334; doi:10.1186/s12870-017-1217-x)

A.

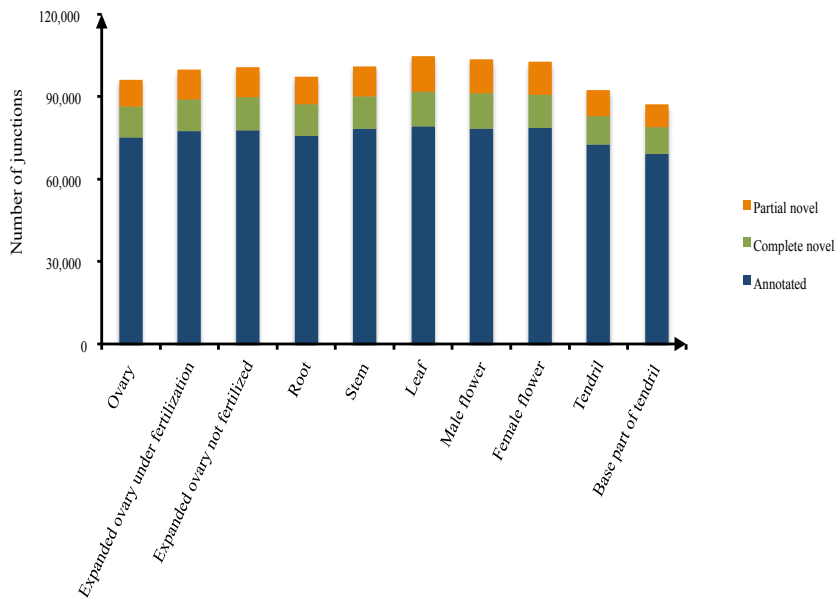

B.

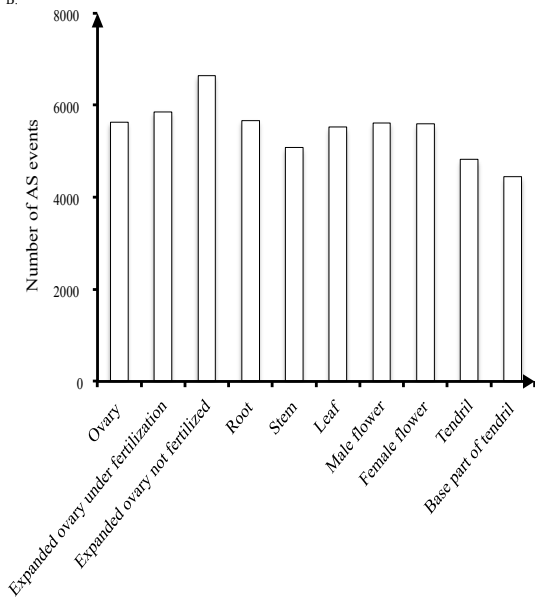

C.

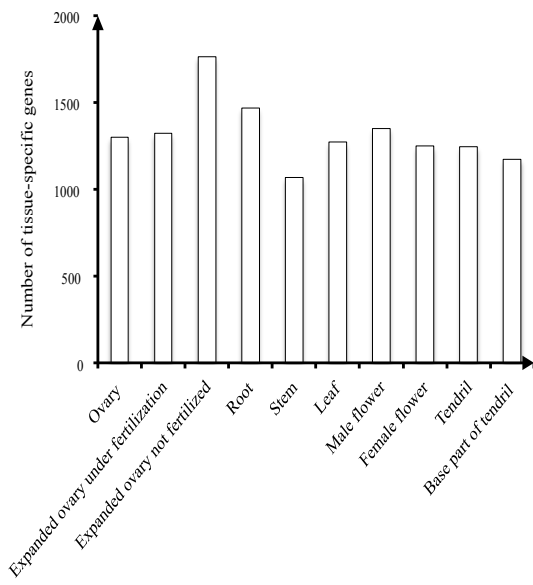

Supplement: Additional file 1: Figure S1. — AS events of ten tissues. (a) Numbers of junctions. The splice sites were divided into the 5′splice site (5’SS) and 3′splice site (3’SS). The dark blue colour represents the number of junctions with two splice sites have been annotated in genome annotation. The green colour represents the number of junctions whose two splice sites are novel in genome annotation. The orange colour represents the number of junctions whose splice sites were found in genome annotation. (b) Number of AS events. (c) Number of genes with tissue-specific AS. (PDF 766 kb) [file 12870_2017_1217_MOESM1_ESM.pdf]

A.

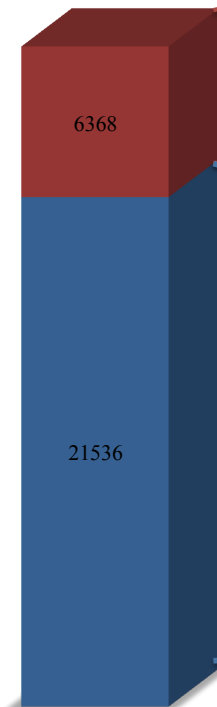

B.

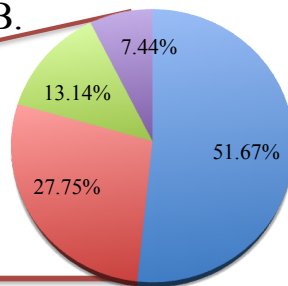

C.

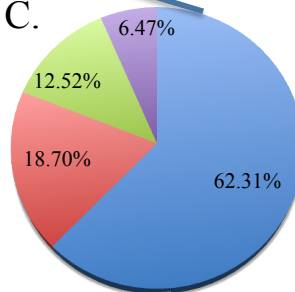

- Among tissue
- Within tissue
- IR
- AA
- AD
- ES

Supplement: Additional file 2: Figure S2. — AS events of within-tissue and among-tissue. (a) The total number of 4 basic types of AS events for among-tissue and within-tissue. (b) Basic types of AS events among tissues. (c) Basic types of AS events within tissues. (PDF 488 kb) [file 12870_2017_1217_MOESM2_ESM.pdf]

A.

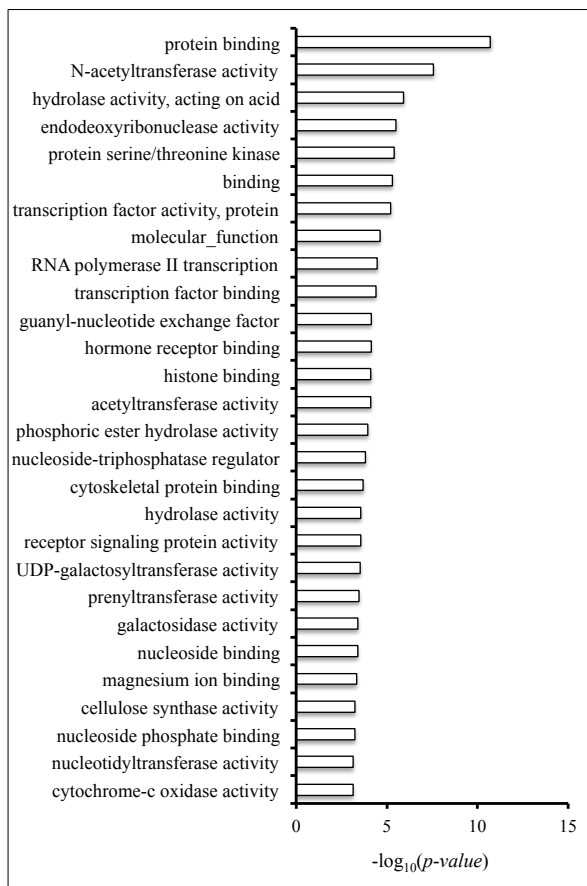

B.

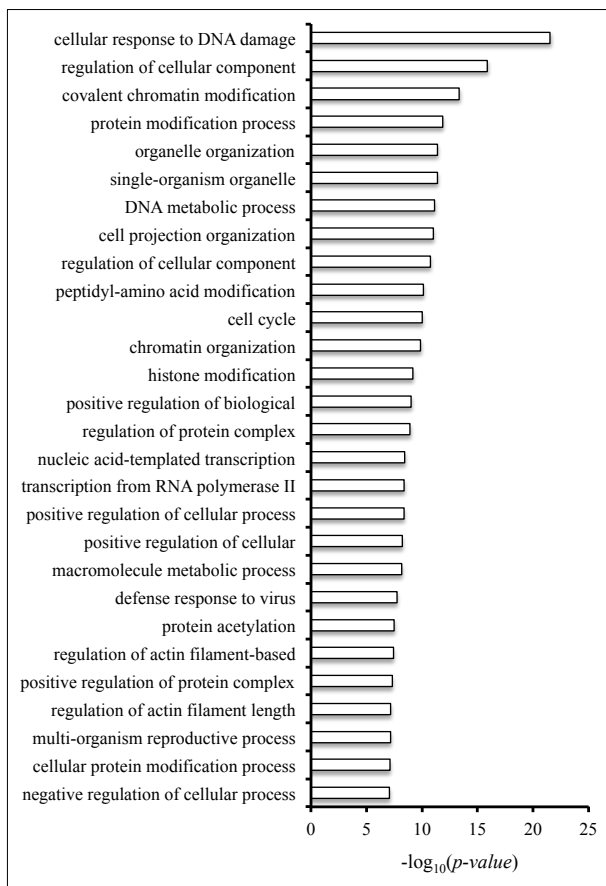

Supplement: Additional file 3: Figure S3. — GO enrichment of AS genes. Only the top 30 significantly enriched GO terms are listed. (a) Molecular function. (b) Biological process. (PDF 298 kb) [file 12870_2017_1217_MOESM3_ESM.pdf]

A.

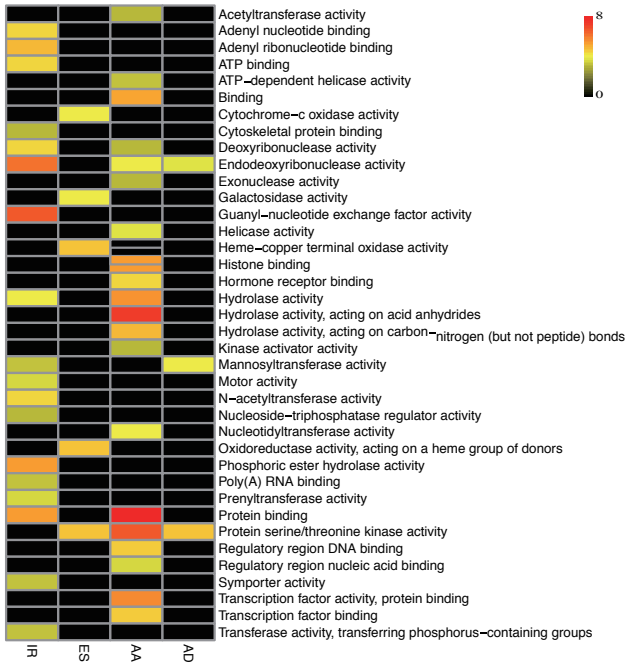

B.

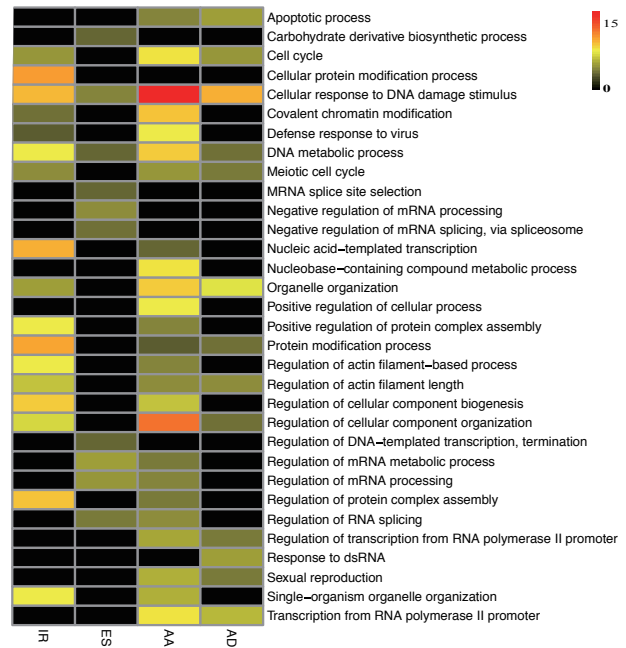

Supplement: Additional file 4: Figure S4. — GO enrichment analysis of the basic AS types. Colour squares represent $-{\log}_{10}^{p- value}$−log10p−value for the enrichment of GO terms. Red means that the term is significantly enriched, and black represent no enrichment in this term. Only significant enrichment GO terms of each type were listed. (a) Molecular function. (b) Biological process. (PDF 969 kb) [file 12870_2017_1217_MOESM4_ESM.pdf]

A.

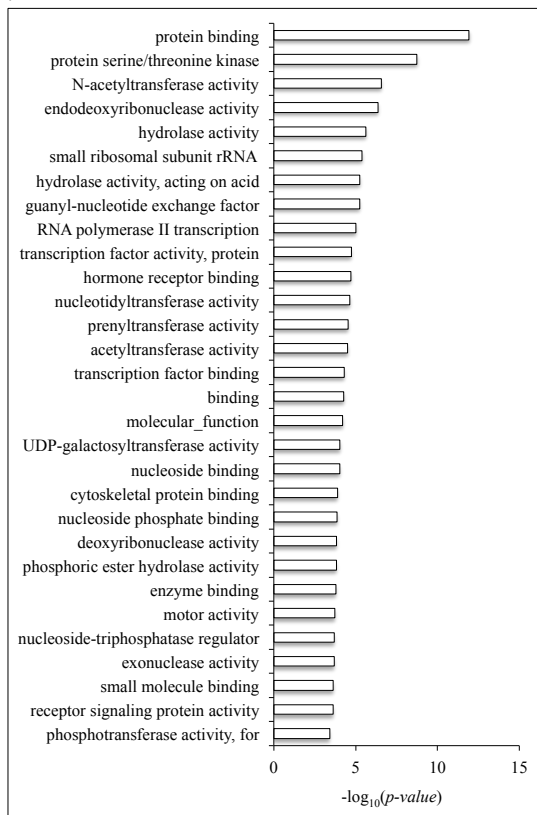

B.

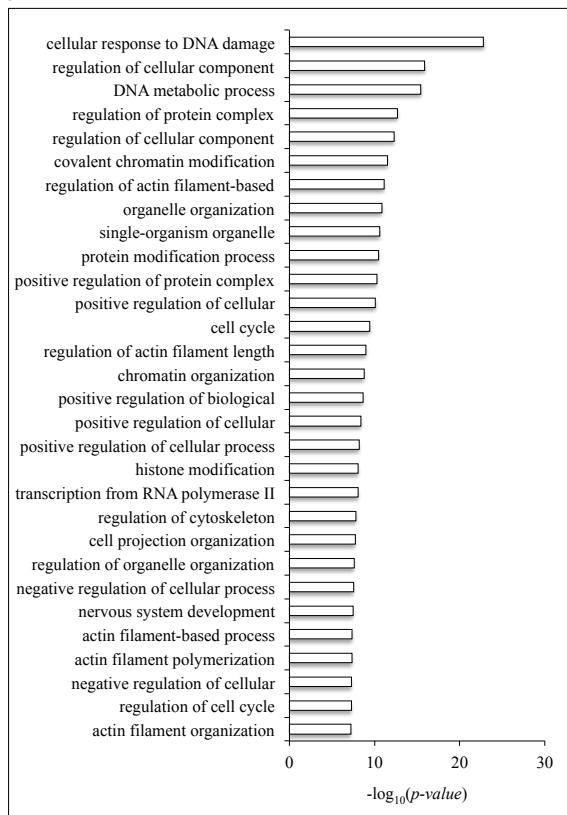

Supplement: Additional file 5: Figure S5. — GO enrichment of genes with tissue-specific AS. Only the top 30 significant enriched GO term were listed. (a) Molecular function (b) Biological process. (PDF 305 kb) [file 12870_2017_1217_MOESM5_ESM.pdf]

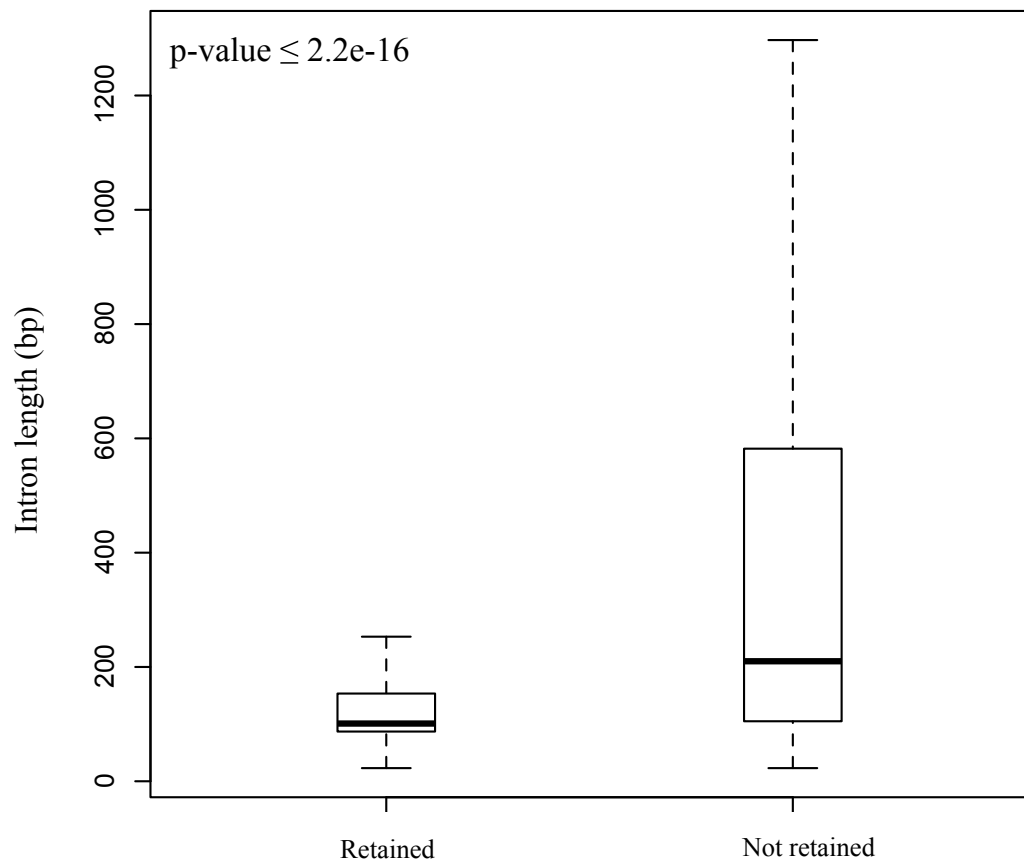

Supplement: Additional file 6: Figure S6. — The lengths of introns between the retained introns and not retained introns. (PDF 102 kb) [file 12870_2017_1217_MOESM6_ESM.pdf]

A.

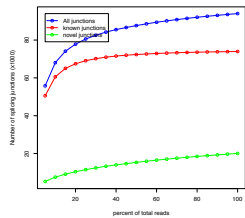

B.

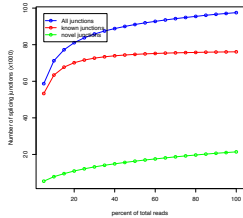

C.

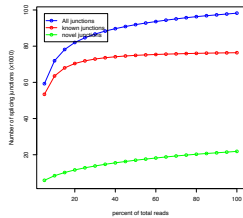

D.

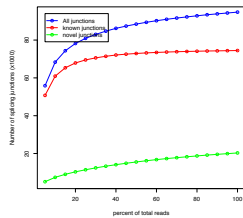

E.

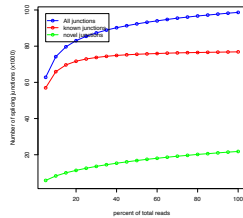

F.

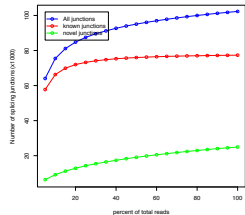

G.

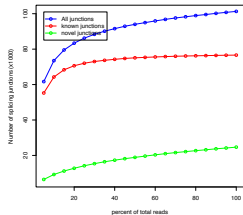

H.

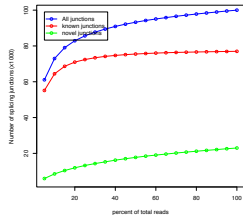

I.

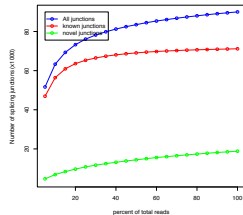

J.

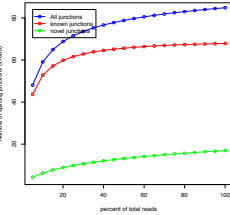

Supplement: Additional file 9: Figure S9. — Saturation analysis of junction detection. (a) Ovary. (b) Expanded ovary under fertilization. (c) Expanded ovary not fertilized. (d) Root. (e) Stem. (f) Leaf. (g) Male flower. (h) Female flower. (i) Tendril. (j) Base part of tendril. (PDF 570 kb) [file 12870_2017_1217_MOESM9_ESM.pdf]
