# Supplementary material for: The comparison of alternative splicing among the multiple tissues in cucumber
Source: BMC Plant Biol. 2018 Jan 5;18:5. doi: 10.1186/s12870-017-1217-x (PMC5755334; doi:10.1186/s12870-017-1217-x)
Supplement: Additional file 7: Figure S7. — Verify of AS events by RT-PCR in leaf. (PDF 759 kb) [file 12870_2017_1217_MOESM7_ESM.pdf]

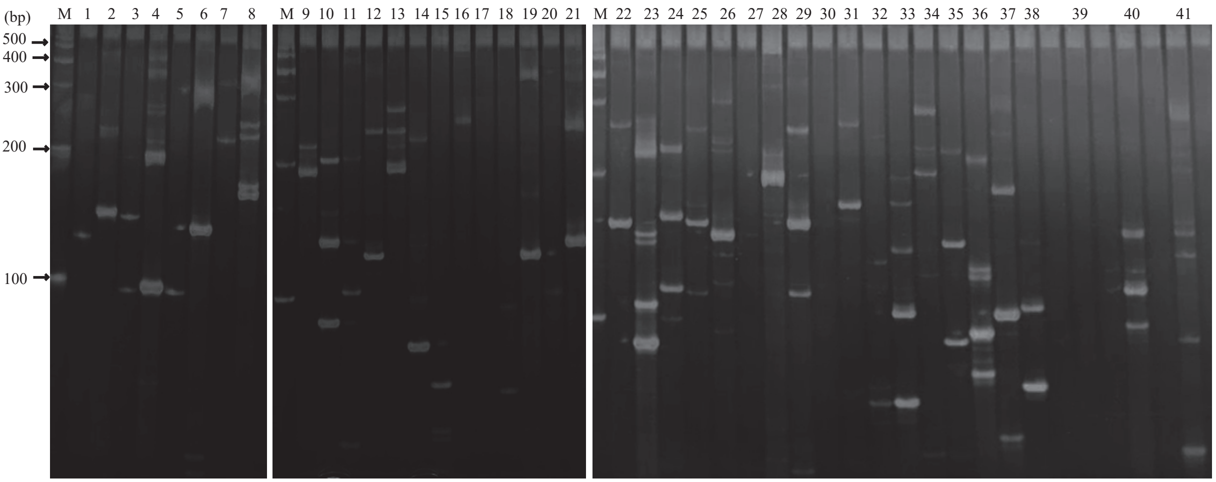


| No. | Gene | AS sites | Length of product (bp) | Forward primer | Reverse primer | AS state |
|---|---|---|---|---|---|---|
| 1 | Csa2G011460 | 2039351~2039486 | 120~285 | TGTACCTAGTTCAACCGTCGAT | CTCCTGATAAGTGTACGACCCA | 1[1] |
| 2 | Csa1G185100 | 11244000,11244076~ | 140~217 | TTTCTCCCTCTGGCAGCC | GCTGGCCTTGACAACAGG | 1 |
| 3 | Csa1G153010 | 9933285~9933326 | 97~136 | ACTGAAGACTGAACCTAACTCCA | TTGGTTCAATCAATTCCCACCC | 1 |
| 4 | Csa1G423220 | 15498451,15498543~ | 100~192 | TGGAGGGTTCAACAGGCC | CCTCTCCAGCAAAGTCAAGC | 1 |
| 5 | Csa2G351790 | 16226745,16226985~ | 87~327 | GTAGTCGTCTGGTTCTCTTTGC | GCGATTCGTACTGGATGATGAG | 1 |
| 6 | Csa1G526830 | 18380225,18380385~ | 131~291 | TCAATGTTGGAGTGCTTTTGGT | CCATTTCCCCTCTTCCGTTTAC | 1 |
| 7 | Csa4G296280 | 12088302,12088404~ | 213~315 | GGATTCTTGGAAGGAAGTCTGC | GTAACTGAGACACAATGAGCCC | 1 |
| 8 | Csa1G528590 | 18582349,18582523~ | 160~334 | GAAGGAGAAAGGGTTGGAAGTG | ATCTCAGCCTGACCAGTTAACT | 1 |
| 9 | Csa3G238180 | 15315079,15315185~ | 187~293 | ATGTACCAAGCGCTGAAGAGAA | TCATCGATTGTTAAGTACCCATCT | 0[2] |
| 10 | Csa1G153010 | 9933285~9933326 | 89~128 | CCTCTGGGTTTGGGTGAAGA | GCTGGTCTTGACTTTAGGCA | 1 |
| 11 | Csa3G167360 | 11105552,11105649~ | 103~200 | TTCCTCCAGAGTTGTTTGTTGG | CCGACGTTAACCACTACAATCC | 1 |
| 12 | Csa1G118890 | 9015945,9016057~ | 122~234 | TCCTCGAATCCCAAAATGCATG | GTGTTTATCTGTGTCTGTGGGG | 0 |
| 13 | Csa3G129690 | 8363337,8363448~ | 191~302 | TGGAATCTTTGCCAAGGATCAT | TGGGAACTAAATACACCTGGCT | 0 |
| 14 | Csa1G108810 | 8775996~8776133 | 79~216 | CACTAGTTTCGCACATCCGG | AGGAGACGAAGCCATGGAAA | 1 |
| 15 | Csa2G431120 | 22444941~22444950 | 68~77 | CCGAGAAGCTTCAAATTCGC | GTTTCCACCACCTTCTTCAGT | 0 |
| 16 | Csa3G209450 | 14188373~14188624 | 247~498 | AGCACGAGAGGAAAGCGA | CTGCTCAGCCAAGGTTGC | 1 |
| 17 | Csa2G379220 | 19251088~19251099 | 72~83 | CGTTGAGGCTCGAGGGATT | TTGACAGCCATCTTCAACGC | 0 |
| 18 | Csa3G563280 | 22093767~22093797 | 65~85 | TCCTTACATCTCTCTCCGCA | TCCCACACAAAACACCCCA | 1 |
| 19 | Csa2G351790 | 16226745,16226985~ | 121~361 | CGTCTGGTTCTCTTTGCACC | ACCCGCCTGACATACCTG | 1 |
| 20 | Csa3G836450 | 33381355~33381388 | 100~133 | TGTTGGCAGTGTTGAAGAGG | ATCCACAGCTGCTCTCCC | 1 |
| 21 | Csa2G279220 | 13362969,13363079~ | 128~238 | GGTGGACCCTAGGCTGTAAATA | GCCTGCCTCTTCATAACATTGA | 1 |
| 22 | Csa3G566330 | 22208318,22208420~ | 152~254 | GCGCTCATATATGTAGTCCTGG | AATGGTCGAGCAAATACAGAGC | 1 |
| 23 | Csa6G290880 | 14057609~14057625 | 89～105 | AAAGCAGAGCAGGACAGTGA | AGATTCATTGTTTCTGGATGCCA | 1 |
| 24 | Csa3G702580 | 26645751~26645794 | 114~157 | GCTTCCCCAAATGACAGACC | TGTAGAGGGCATGTCAGGTG | 1 |
| 25 | Csa6G499000 | 24607459,24607500~ | 111~152 | GAGAAGTCACTGGAGTCTGGAT | GCCTTACGCCATTCATTTTCTC | 1 |
| 26 | Csa3G914040 | 39654639,39654786~ | 146~291 | ATGACGATGACAGACGAGAAGA | AATTTCTTCACAATCGCAACCG | 1 |
| 27 | Csa3G002670 | 427843,427976~ | 168~301 | AAAACATGACACCACCCTTTGT | TTCCTAACTGTTGTCGCATTCC | 1 |
| 28 | Csa4G294930 | 11792217,11792597~ | 191~571 | GTCCATCAGTTAAAGCACGTCT | CTCAATGTTGGTCAAAGAAAAGGT | 1 |
| 29 | Csa6G525640 | 28584255,28584385~ | 114~244 | CGAGAGGGGAAATTGGGAAA | GGCTTGTCCGAGAATGTGC | 0 |
| 30 | Csa4G420140 | 15806683~15806704 | 67~88 | CCATCAATTGCACCATCTCCA | GTGTGAATCCCCATTGAGTGA | 0 |
| 31 | Csa7G238950 | 8692089,8692181~ | 168~260 | GACAGCATCAATCGCACTCTTT | AGGGTCAATTGATGGATCTGGT | 1 |
| 32 | Csa4G669240 | 23378395~23378400 | 70~75 | CCACCAACATGCCCTTACTA | CCCATCTGATTGCGCCAG | 0 |
| 33 | Csa7G324110 | 11257298~11257330 | 70～102 | CGTTGGTGAGATCGCGC | TCAAGTATTCATCTTCTGCCCTC | 1 |
| 34 | Csa5G524790 | 18618772,18618858~ | 193~279 | ATGTGCACGTCAGTTGATTGAA | TCTTTTCTGTCCCCAAAGCAAA | 1 |
| 35 | Csa7G352440 | 12621289~12621337 | 90～138 | GGTTGTTTATTCCCCATGTTTGC | ATCTTTACGTCGTGCACAATGT | 1 |
| 36 | Csa5G602190 | 22135860~22135875 | 78~93 | TTGTGATGTTGAACAGGCCG | ATAGAAACCCCAGGCAAGCA | 1 |
| 37 | Csa7G374660 | 13602239~13602317 | 102～180 | GGAACTGGAGGGAGGTTTCA | TGGCCATCTGAAACCCCAAT | 0 |
| 38 | Csa5G621940 | 24495360~24495390 | 74~104 | GTTCTTGACTACGAAGCTTCCC | TTGGTCGATGTTTTGAACCTCC | 0 |
| 39 | Csa5G623710 | 24965476,24965579~ | 100~203 | GTTCTTGACTACGAAGCTTCCC | TTGGTCGATGTTTTGAACCTCC | 1 |
| 40 | Csa6G011660 | 1241245~1241261 | 96~112 | TCAAGTCTTGCAGGTGTGGA | CGTCTACAAGCATGGCGTC | 1 |
| 41 | Csa6G151120 | 10632399,10632510~ | 151~262 | AAAGTTGCAGAGCTCCTTCTTG | TGGGTTCTATCAGGTCATGGTT | 1 |

[1] AS event predicted in leaf. [2] no AS events predicted in leaf.
