# Supplementary material for: The comparison of alternative splicing among the multiple tissues in cucumber
Source: BMC Plant Biol. 2018 Jan 5;18:5. doi: 10.1186/s12870-017-1217-x (PMC5755334; doi:10.1186/s12870-017-1217-x)
Supplement: Additional file 8: Figure S8. — Verify of AS events by RT-PCR in leaf and stem. (PDF 2393 kb) [file 12870_2017_1217_MOESM8_ESM.pdf]

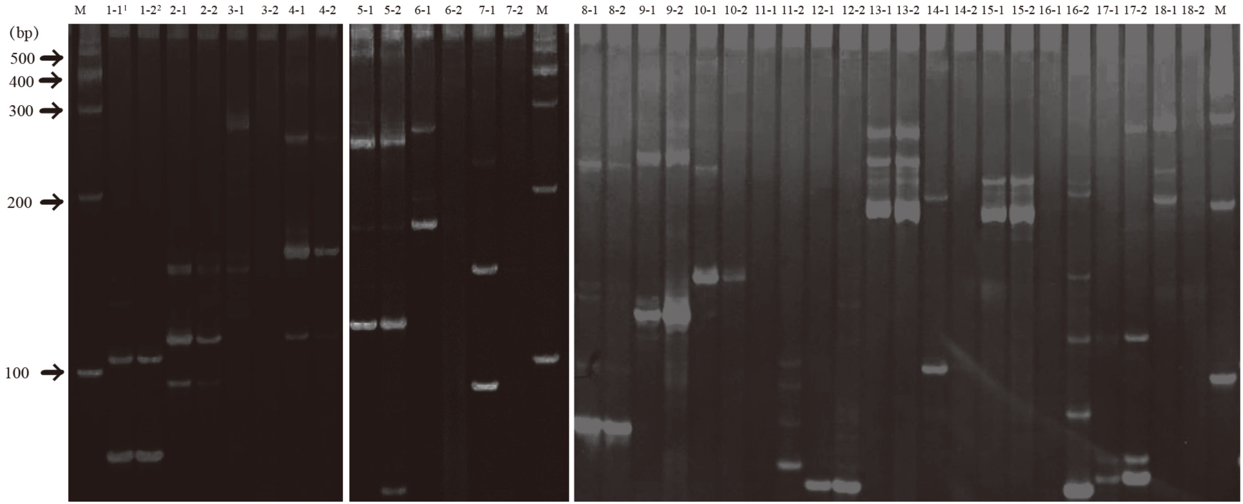

[1] Products of RT-PCR in the leaf. [2] Products of RT-PCR in the stem

No.	Gene	AS sites	Length of product (bp)	AS state in leaf	AS state in stem
1	Csa5G621940	24495360~24495390	74~104	0	1[1]
2	Csa6G011660	1241245~1241261	96~112	1	0[2]
3	Csa6G151120	10632399,10632510~	151~262	1	0
4	Csa6G499000	24607459,24607500~	111~152	1	0
5	Csa6G525640	28584255,28584385~	114~244	0	1
6	Csa7G238950	8692089,8692181~	168~260	1	0
7	Csa7G352440	12621289~12621337	90～138	1	0
8	Csa1G108810	8775996~8776133	79~216	1	0
9	Csa1G118890	9015945,9016057~	122~234	0	1
10	Csa1G185100	11244000,11244076~	140~217	1	0
11	Csa2G379220	19251088~19251099	72~83	0	1
12	Csa2G431120	22444941~22444950	68~77	0	1
13	Csa3G129690	8363337,8363448~	191~302	0	1
14	Csa3G167360	11105552,11105649~	103~200	1	0
15	Csa3G238180	15315079,15315185~	187~293	0	1
16	Csa4G420140	15806683~15806704	67~88	0	1
17	Csa4G669240	23378395~23378400	70~75	0	1
18	Csa5G524790	18618772,18618858~	193~279	1	0

[1] AS event predicted in this tissue. [2] no AS events predicted in this tissue.
